# Overexpression of IL-10 Enhances the Efficacy of Human Umbilical-Cord-Derived Mesenchymal Stromal Cells in *E. coli* Pneumosepsis

**DOI:** 10.3390/jcm8060847

**Published:** 2019-06-13

**Authors:** Mirjana Jerkic, Claire Masterson, Lindsay Ormesher, Stéphane Gagnon, Sakshi Goyal, Razieh Rabani, Gail Otulakowski, Haibo Zhang, Brian P. Kavanagh, John G. Laffey

**Affiliations:** 1 St. Michael’s Hospital, Keenan Research Centre for Biomedical Science, University of Toronto, Toronto, ON M5B 1T8, Canada; jerkicm@smh.ca (M.J.); lormeshe@mail.uoguelph.ca (L.O.); GagnonS@smh.ca (S.G.); sakshigoyal383@gmail.com (S.G.); Razieh.Rabani@utoronto.ca (R.R.); Zhangh@smh.ca (H.Z.); 2 Regenerative Medicine Institute (REMEDI) at CÚRAM Centre for Research in Medical Devices, Biomedical Sciences Building, National University of Ireland Galway, Galway H91 TK33, Ireland; claire.masterson@nuigalway.ie; 3 Department of Critical Care Medicine, Hospital for Sick Children, Toronto, ON M5G 1X8, Canada; Gail.Otulakowski@sickkids.ca (G.O.); Brian.Kavanagh@sickkids.ca (B.P.K.); 4 Departments of Anesthesia, Physiology and Interdepartmental Division of Critical Care, University of Toronto, Toronto, ON M5G 1E2, Canada; 5 Anaesthesia, School of Medicine, National University of Ireland, Galway H91 TK33, Ireland

**Keywords:** mesenchymal stromal cell, pneumonia, sepsis, macrophage, phagocytosis

## Abstract

Enhancing the immunomodulatory effects of mesenchymal stromal cells (MSCs) may increase their effects in sepsis. We tested the potential for overexpression of Interleukin-10 (IL-10) in human umbilical cord (UC) MSCs to increase MSC efficacy in *Escherichia coli* (*E. coli*) pneumosepsis and to enhance human macrophage function. Pneumonia was induced in rats by intratracheal instillation of *E. coli* ((2.0–3.0) × 10^9^ Colony forming units (CFU)/kg). One hour later, animals were randomized to receive (a) vehicle; (b) naïve UC-MSCs; or (c) IL-10 overexpressing UC-MSCs (1 × 10^7^ cells/kg). Lung injury severity, cellular infiltration, and *E. coli* colony counts were assessed after 48 h. The effects and mechanisms of action of IL-10 UC-MSCs on macrophage function in septic rodents and in humans were subsequently assessed. Survival increased with IL-10 (9/11 (82%)) and naïve (11/12 (91%)) UC-MSCs compared to vehicle (9/15 (60%, *p* = 0.03). IL-10 UC-MSCs—but not naïve UC-MSCs—significantly decreased the alveolar arterial gradient (455 ± 93 and 520 ± 81, mmHg, respectively) compared to that of vehicle animals (544 ± 52, *p* = 0.02). Lung tissue bacterial counts were significantly increased in vehicle- and naïve-UC-MSC-treated animals but were not different from sham animals in those treated with IL-10 overexpressing UC-MSCs. IL-10 (but not naïve) UC-MSCs decreased alveolar neutrophils and increased alveolar macrophage percentages compared to vehicle. IL-10 UC-MSCs decreased structural lung injury compared to naïve UC-MSC or vehicle therapy. Alveolar macrophages from IL-10-UC-MSC-treated rats and from human volunteers demonstrated enhanced phagocytic capacity. This was mediated via increased macrophage hemeoxygenase-1, an effect blocked by prostaglandin E2 and lipoxygenase A4 blockade. IL-10 overexpression in UC-MSCs enhanced their effects in *E. coli* pneumosepsis and increased macrophage function. IL-10 UC-MSCs similarly enhanced human macrophage function, illustrating their therapeutic potential for infection-induced acute respiratory distress syndrome (ARDS).

## 1. Introduction

Mesenchymal stromal cells (MSCs) represent a promising therapeutic strategy for sepsis [1] and for acute respiratory distress syndrome (ARDS) [2]. MSCs are multipotent adult stromal cells with the capacity to differentiate into several cell types and are present in multiple tissues, including the bone marrow, adipose tissue, and umbilical cord. They are found in the perivascular space in several tissues, including the umbilical cord, which is an easily accessible biologic waste product that generates high amounts of MSCs. We recently demonstrated that human umbilical-cord-derived MSCs (UC-MSCs) attenuate *Escherichia coli* (*E. coli*)-induced acute lung injury [3].

MSCs have shown substantial therapeutic promise in relevant preclinical models [4,5,6,7,8] of pneumonia, ARDS, and sepsis. The immune modulatory effects of MSCs are mediated via both cell-to-cell contact-dependent and -independent (i.e., paracrine) mechanisms [1,2]. A key area of investigation is the potential to further enhance the immunomodulatory effects of MSCs to increase their therapeutic potential [9]. Inteleukin-10 (IL-10) is a key immunoregulatory cytokine that can suppress potentially damaging pro-inflammatory responses, aid injury resolution, and restore tissue function in both ARDS and sepsis [10].

The immunomodulatory activities of IL-10 may interact favorably with those of MSCs to produce enhanced effects. Direct administration of IL-10 has been demonstrated to attenuate hyperoxia-induced lung injury [11]. Overexpression of IL-10 has been demonstrated to reduce ischemia–reperfusion injury in lung transplant models [12]. Prior studies of overexpression of IL-10 in MSCs has demonstrated enhanced MSC therapeutic efficacy in preclinical models of endotoxin-induced [13] and ischemia–reperfusion-induced [14] lung injury and following myocardial infarction [15]. The effects of IL-10 overexpressing MSCs in the setting of live bacterial infection are not known.

These studies investigated whether IL-10 overexpression could enhance UC-MSC efficacy in the setting of live bacterial pneumonia-induced ARDS. We hypothesized that IL-10 overexpressing UC-MSCs would more effectively attenuate *E. coli*-induced lung injury compared to naïve UC-MSCs. We further hypothesized that this effect would be mediated in part via enhanced macrophage phagocytosis and killing of *E. coli* bacteria.

## 2. Experimental Section

All work was approved by the Animal Care and Use Committee of the Keenan Research Centre for Biomedical Science, St Michael’s Hospital, Toronto (ACC648) and conducted under license from Health Canada. Specific-pathogen-free adult male Sprague Dawley rats (Charles River Laboratories, Senneville, Quebec, QC, Canada) weighing between 350 and 450 g were used in all experiments. The studies on human peripheral blood mononuclear cells were approved by the Research Ethics Board of St Michael’s Hospital, Toronto (REB: 14-278).

### 2.1. Human Mesenchymal Stromal Cells

Human umbilical cords were obtained from full-term, consenting donors undergoing caesarean section at Mount Sinai Hospital, Toronto, Canada using the protocol approved by research ethics boards at both the University of Toronto and Mount Sinai Hospital’s Research Centre for Women’s and Infants’ Health (RCWIH). UC-MSCs were non-enzymatically extracted by a proprietary methodology and provided by Tissue Regeneration Therapeutics (TRT) Inc., Toronto, ON, Canada, as previously described [16,17]. Briefly, following removal of the umbilical cord epithelium using blunt dissection, the three cord vessels were separated and then digested in 100 U/mL type I collagenase and 0.01 U/mL hyaluronidase for 3–5 h. The resulting suspension was centrifuged at 285× *g* for 10 min, the supernatant aspirated, the cell pellets pooled, and erythrocytes lysed using 0.8% ammonium chloride (0.8%). The tube was then centrifuged for 10 min at 285× *g*, the supernatant was discarded, and the cells were washed and counted. Cells harvested by this process were cultured in Lonza TheraPEAK™ MSCGM-CD serum-free complete medium (Cedarlane, Burlington, ON, Canada) supplemented with antibiotics (28 µM penicillin G, 104 µM gentamycin, and 324 nM amphotericin B) on fibronectin-coated flasks, passaged twice, and cryopreserved. Routine flow cytometry on identically processed lots revealed highly positive expression of CD90, CD73, CD105, CD140b, and CD166 and a lack of CD31, CD34, CD45, and MHC-II in naïve and IL-10 UC-MSCs (Figure S2). The adipogenic, osteogenic, and chondrogenic differentiation potential of these cells was previously reported by our group [3]. Cryopreserved UC-MSCs (passage 2) were thawed, and 1 million cells per T175 flask (6000/cm^2^) were seeded and cultured for 3–5 days in serum-free complete medium. These were lifted from the flask on the day of administration using 10 mL of TrypLE™ Express (ThermoFisher Sci., Burlington, ON, Canada), then washed with 1× phosphate-buffered saline (PBS) by centrifugation, counted, and resuspended in PBS vehicle for delivery. An IL-10 recombinant adenovirus was purchased (VH869610, Vigene Biosciences, Rockville, MD, USA). UC-MSCs were seeded at a cell density of 23,000 cells/cm^2^ and 24 h later were exposed to the virus at a multiplicity of infection (MOI) of 100 in 100 μL/cm^2^ of culture medium. After 24 h of incubation at 37 °C and 5% CO_2_, cells were washed with PBS three times, fresh growth medium was added, and the UC-MSCs were harvested 3 days later for experimental use.

### 2.2. E. coli Pneumonia Model

*E. coli* with serotype O6, Biotype 1 was obtained from American Type Culture Collection (ATCC^®^ 25922, Manassas, VA, USA) and used in these experiments. Preliminary experiments were performed to determine the bacterial load of intratracheal *E. coli* required to produce a lung injury over a 48 h period. Animals were anesthetized by inhalational induction with isoflurane and intraperitoneal 40 mg/kg ketamine (Pfizer, Kent, UK). After confirmation of depth of anesthesia by paw clamp, intravenous access was obtained via tail vein, laryngoscopy was performed, and the animals were intubated with a size 14G intravenous catheter (BD Insyte^®^, Becton Dickinson Ltd., Oxford, UK). A quantity of (2–3) × 10^9^ *E. coli* CFU in a 300 µL PBS suspension was instilled into the lungs via the trachea using a 1 mL syringe, and the animals were allowed to recover [18].

One hour following intratracheal instillation of *E. coli* bacteria, animals were randomized to intravenous administration of (a) vehicle (PBS, 300 µL); (b) naïve UC-MSCs (1 × 10^7^ cells/kg); or (c) IL-10 overexpressing UC-MSCs (IL-10 UC-MSCs, 1 × 10^7^ cells/kg). Animals were subsequently allowed to recover and housed. The effects on physiologic indices of lung injury, cellular infiltration, and *E. coli* colony counts in bronchoalveolar lavage were assessed after 48 h.

Forty-eight hours after *E. coli* instillation, animals were re-anesthetized as described above; intravenous access was established via tail vein and anesthesia was maintained with alfaxalone (alfaxadone 0.9% and alfadadolone acetate 0.3%). A tracheostomy tube was inserted, and intra-arterial access was sited in the carotid artery. Muscle relaxation was induced with cisatracurium besylate, and the lungs were mechanically ventilated using a small animal ventilator (CWE SAR 830 AP, CWE Inc., Pennsylvania, PA, USA) with 30% O_2_ in 70% N_2_ at a respiratory rate of 80 min^−1^, tidal volume of 6 mL·kg^−1^, and positive end-expiratory pressure of 2 cm H_2_O as previously described [19,20]. The systemic arterial blood pressure and peak airway pressure were continually measured. Body temperature was maintained at 36–37.5 °C. Lung static compliance and arterial blood gas analysis were measured after 20 min and were repeated on 100% O_2_ after 15 min [19].

### 2.3. Ex Vivo Analyses

Animals were then euthanized by exsanguination under anesthesia. The heart–lung block was dissected from the thorax, bronchoalveolar lavage (BAL) was performed, and the BAL fluid differential, leukocyte counts, and lung bacterial colony counts were completed. The BAL fluid was centrifuged, and the supernatant was snap frozen and stored at −80 °C. The BAL concentrations of tumor necrosis factor alpha (TNF-α), IL-6, monocyte chemoattractant protein-1 (MCP-1), and IL-10 were determined using enzyme-linked immunosorbent assay (ELISA) (R & D Systems, Minneapolis, MN, USA), and the BAL protein concentrations were measured (Bio-Rad protein assay, Hercules, CA, USA). The left lung was isolated and fixed [21], and the extent of histologic lung damage was determined using quantitative stereological techniques. A portion of the right lung lobes was snap frozen for further analyses, and the remainder was homogenized and assessed for *E. coli* colony counts. All ex vivo analyses (BAL analyses, histologic analyses) were performed by blinded investigators.

Western blot analysis was performed according to an established protocol [22]. Briefly, tissues were homogenized in TNE buffer (0.05 M Tris/HCl, pH 7.4, 0.1 M NaCl, 1 mM of Ethylenediaminetetraacetic acid-EDTA) supplemented with 1% Triton X-100 and protease/phosphatase inhibitors, and equal protein amounts were fractionated on 4%–12% gradient Novex^®^ Bis-Tris Gels for protein separation (NuPAGE) gels (Invitrogen, Burlington, ON, Canada) and transferred to a polyvinylidene difluoride (PVDF) membrane (Immobilon-P, Millipore Corp, Bedford, MA, USA). After blocking with 5% milk in Tris-Buffered Saline and Tween 20 (TBS-T), the blot was incubated with primary antibody for 2 h (β-actin) or overnight (heme oxygenase-1 (HO-1)), followed by a secondary antibody conjugated with horseradish peroxidase for 1 h. The following primary antibodies were used: HO-1 (HSP-32), 1:1000 (mouse IgG1; Enzo Life Sci, Farmingdale, NY, USA). Signals were detected using an ECL-Plus kit (Amersham Biosci, Piscataway, NJ, USA). Band intensities were quantified and expressed relative to that of β-actin, 1:10,000 (mouse IgG1, Sigma-Aldrich, Oakville, ON, Canada).

### 2.4. Assessment of Macrophage Function

Lung/blood monocyte-derived macrophages (Mф) were isolated from sham and *E. coli*-injured animals that received naïve and IL-10 overexpressing UC-MSC or vehicle therapy by Ficoll gradient. Mф were then seeded (50,000 cells/well) and left overnight without treatment. The phagocytic capacity and phagosomal superoxide production were determined using the Zymosan assay [23] (four experiments done in duplicate), as described previously [23]. Briefly, Alexa-488-conjugated serum opsonized zymosan (SOZ; Sigma-Aldrich, Oakville, ON, Canada) was added to cells growing on 18 mm coverslips in 12-well plates at the concentration of 0.125 mg/mL. The 12-well plate was then centrifuged for 1 min (1500 rpm) to rapidly bring the SOZ in contact with the cells. The cells were then incubated at 37 °C for 30 min. Excess particles were removed by three washes with PBS, and the cells were fixed in 4% paraformaldehyde in PBS for 15 min. The coverslips were mounted on the slides using mounting medium (Daco Manufacturing Ltd., Woodbridge, ON, Canada) and stored under dark cold conditions. Cells were visualized by confocal microscopy using a laser scanning Zeiss LSM700 microscope equipped with a single pinhole (Carl Zeiss Microscopy GmbH, Peabody, MA, USA) and ZEN software (2012 blue edition). Counting of engulfed (green) particles was done in 8–10 randomly chosen fields per slide using Image-J (version 1.48a, NIH, USA) software. The phagocytic index was calculated as the average number of particles ingested per phagocyte. The roles of Prostaglandin E2 (PGE2) and Lipoxin A4 (LXA4) in HO-1 expression and the HO-1-mediating effects of naïve and Il-10 overexpressing hUC-MSCs on human macrophage phagocytosis were assessed using a PGE2 inhibitor (10 µM, CAY10526) and a 15-lipoxygenase (15-LO) inhibitor (2 µM, PD146176), respectively.

### 2.5. Statistical Analysis

Data were analyzed using GraphPad Prism (GraphPad^®^ Prism 7.04 software, La Jolla, CA, USA). The distribution of all data was tested for normality using Kolmogorov–Smirnov tests. Data were analyzed by one-way ANOVA with post hoc testing using the Newmann–Keuls Multiple Comparison Test. For survival data analysis the Log Rank test was used. The underlying model assumptions were deemed appropriate on the basis of suitable residual plots. A two-tailed *p* value of <0.05 was considered significant.

## 3. Results

### 3.1. IL-10 UC-MSC Characterization

The naïve UC-MSCs and IL-10 overexpressing UC-MSCs demonstrated the standard surface antigen expression profile (Figure S1) [3]. In addition, the IL-10 UC-MSCs secreted substantially greater amounts of IL-10 in vitro than did naïve UC-MSCs, and this was further enhanced in the presence of macrophages from healthy volunteers and in patients with sepsis (Figure S2). UC-MSC cell morphology (Figure S3) and cell viability (82.4 ± 6.6 vs. 85.3 ± 6.3%) were unchanged in IL-10 overexpressing UC-MSCs compared to in naïve UC-MSCs.

### 3.2. Effects in E. coli Pneumosepsis

#### 3.2.1. Animal Survival

**Figure 1:**
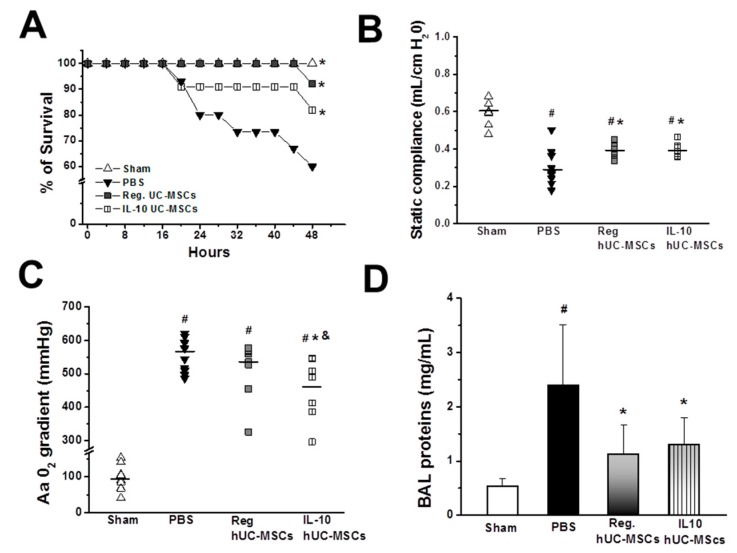
**Human IL-10 umbilical-cord-derived mesenchymal stromal cell (UC-MSC) therapy improved survival and reduced injury severity in *Escherichia coli* pneumonia.** Both naïve and IL-10 UC-MSCs significantly increased the survival of acute respiratory distress syndrome (ARDS) rats (**A**) and improved static lung compliance (**B**); IL-10 overexpressing UC-MSCs—but not naïve UC-MSCs—significantly improved blood oxygenation and alveolar arterial (Aa) gradient compared to vehicle treatment. (**C**) Both naïve and IL-10 UC-MSCs significantly decreased alveolar fluid protein concentrations (**D**) compared to those in vehicle-treated animals. # *p* < 0.05 vs. Sham group; * *p* < 0.05 vs. PBS group; & *p* < 0.05 vs. Regular (Reg.) UC-MSC group. *N* = 9 for all groups except the groups treated with Reg. UC-MSCs (*N* = 11). hUC-MSCs = human umbilical cord mesenchymal stromal cells; BAL = Broncho alveolar lavage; Aa gradient = alveolar arterial gradient.

UC-MSC therapy increased animal survival in *E. coli*-induced pneumonia (Figure 1A), increasing the survival from 60% (9/15) in vehicle-treated animals to 91% (11/12) with naïve UC-MSC therapy and 82% (9/11) in the animals treated with IL-10 UC-MSCs (*p* < 0.05 versus vehicle).

#### 3.2.2. Lung Injury Severity

Both naïve and IL-10 UC-MSCs significantly improved static lung compliance (Figure 1B). IL-10 overexpressing UC-MSCs—but not naïve UC-MSCs—significantly improved blood oxygenation, decreasing the alveolar arterial gradient compared to the vehicle treatment (Figure 1C). Both naïve and IL-10 UC-MSCs significantly decreased alveolar fluid protein concentrations (Figure 1D) compared to those in vehicle-treated animals.

#### 3.2.3. Bacterial Burden

**Figure 2:**
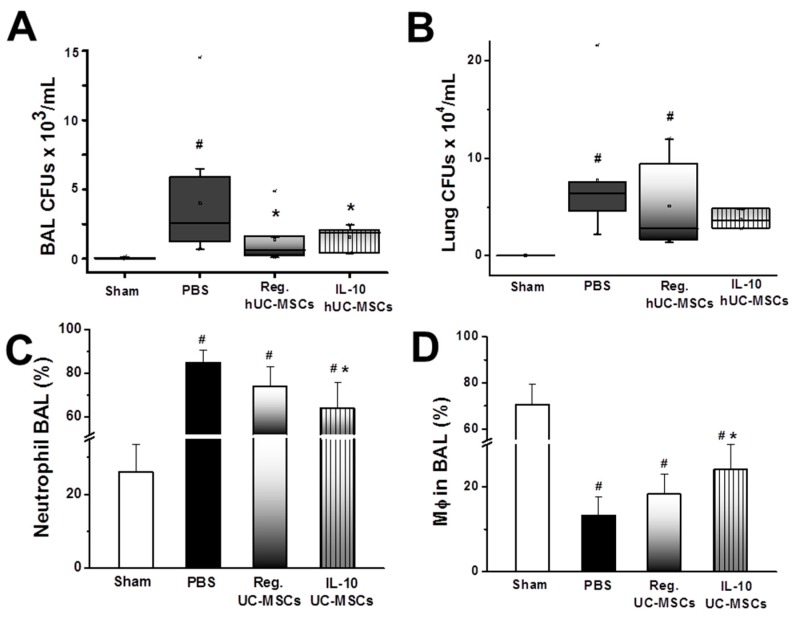
**IL-10 UC-MSCs reduce bacterial load and modulate the innate immune response.** Both IL-10 overexpressing and naïve UC-MSCs reduced alveolar counts of *E. coli* (**A**), while the pulmonary tissue *E. coli* bacterial load in the lungs of the IL-10 UC-MSC group was not significantly different from that in sham rats (**B**); IL-10 overexpressing—but not naïve—UC-MSCs decreased the percentage of alveolar neutrophils (**C**) and increased the percentage of alveolar macrophages following *E. coli* pneumonia (**D**) compared to those in vehicle (PBS)-treated animals. Note: # *p* < 0.05 vs. Sham group; * *p* < 0.05 vs. PBS group; & *p* < 0.05 vs. Regular UC-MSC group. *N* = 9 for all groups except the groups treated with Reg. UC-MSCs (*N* = 11).

Both IL-10 overexpressing and naïve UC-MSCs reduced alveolar counts of *E. coli* (Figure 2A). The pulmonary tissue *E. coli* bacterial load was increased in vehicle- and naïve-MSC-treated animals but was not significantly different in the IL-10 MSC group compared to sham rats (Figure 2B).

#### 3.2.4. Inflammatory Response

**Figure 3:**
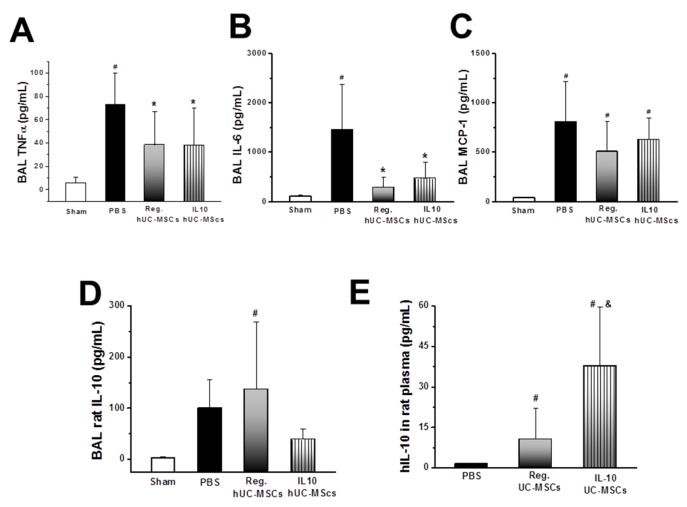
**IL-10 UC-MSC therapy modulated the cytokine response to *E. coli* pneumonia.** Both IL-10 overexpressing and naïve UC-MSCs reduced alveolar concentrations of the pro-inflammatory cytokines TNFα (**A**) and IL-6 (**B**), while the Monocyte chemoattractant protein-1 (MCP-1) decrease was not significant (**C**) when compared to vehicle-treated animals. The concentration of rodent-derived IL-10 was increased only in rats treated with naïve UC-MSCs (**D**). In contrast, the alveolar concentration of human IL-10 was significantly increased in animals treated with IL-10 UC-MSCs when compared to both other groups (**E**), *N* = 9–11 in all groups except Sham group (*N* = 3); # *p* < 0.05 vs. Sham; * *p* < 0.05 vs. Vehicle; & *p* < 0.05 vs. *E. coli* + Reg UC-MSC group.

IL-10 overexpressing—but not naïve—UC-MSCs decreased the percentage of alveolar neutrophils (Figure 2C) and increased the percentage of alveolar macrophages following *E. coli* pneumonia (Figure 2D) compared to those in vehicle-treated animals. Both IL-10 overexpressing and naïve UC-MSCs reduced alveolar concentrations of the pro-inflammatory cytokines TNFα (Figure 3A) and IL-6 (Figure 3B) compared to those in vehicle-treated animals, while the MCP-1 decrease was not significant (Figure 3) The concentration of rodent-derived IL-10 was increased only in rats treated with naïve UC-MSCs (Figure 3D). In contrast, the alveolar concentration of human IL-10 was significantly increased in animals treated with IL-10 UC-MSCs compared to both other groups (Figure 3E).

#### 3.2.5. Structural Lung Injury

**Figure 4:**
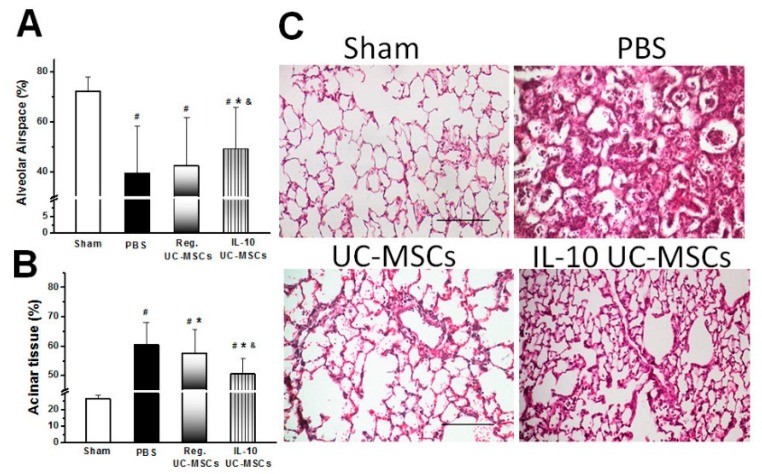
**IL-10 UC-MSC therapy reduced structural lung injury.** Histograms demonstrate that IL-10 UC-MSC—but not naïve UC-MSC—therapy increased the alveolar airspace fraction (**A**) when compared to the vehicle treatment. IL-10 UC-MSC therapy also more effectively attenuated the increase in acinar tissue (**B**) compared to both naïve UC-MSCs and vehicle therapy. Representative photomicrographs of lung from sham, PBS-treated, UC-MSC-treated. and IL-10-UC-MSC-treated (**C**) animals demonstrate reduced lung injury in the naïve and IL-10 UC-MSC-treated groups. The scale bar is 200 micrometers. # *p* < 0.05 vs. Sham group; * *p* < 0.05 vs. PBS group; & *p* < 0.05 vs. UC-MSC group; *N* = 6–7 animals/group (30 fields/animal).

IL-10 overexpressing—but not naïve—UC-MSCs increased the alveolar airspace compared to vehicle therapy (Figure 4A). Both naïve and IL-10 hMSCs reduced the alveolar tissue when compared to vehicle therapy (Figure 4B). IL-10 overexpressing UC-MSCs were more effective than naïve UC-MSCs in reducing alveolar tissue. Photomicrographs of lungs from the sham, vehicle-treated, naïve-UC-MSC-treated, and IL-10 UC-MSC groups are provided in Figure 4C.

### 3.3. Effects on Alveolar Macrophage Function

**Figure 5:**
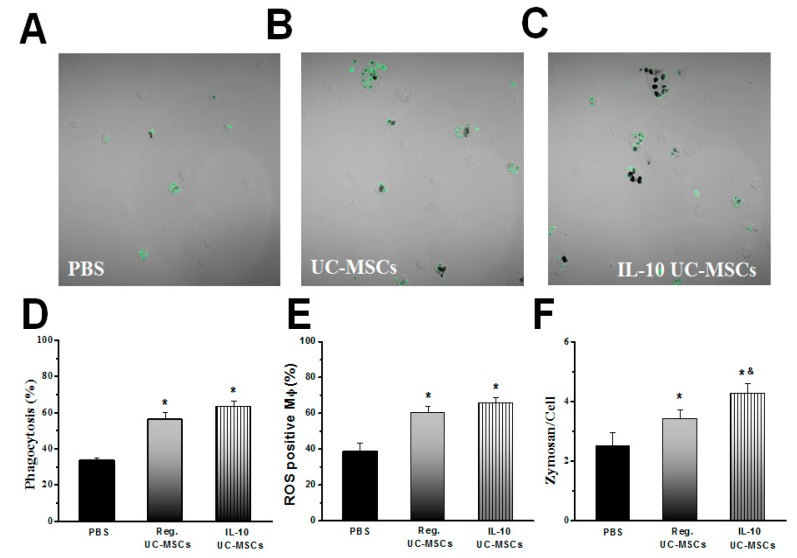
**IL-10 UC-MSC therapy enhances alveolar macrophage function.** Representative photomicrographs of alveolar macrophages isolated from *E. coli* pneumonia rats treated with naïve and IL-10 UC-MSCs demonstrate greater phagocytosis ex vivo than do macrophages from vehicle-treated rats (**Panels A**–**C**), Quantitative analysis confirmed that macrophages isolated from lungs of UC-MSC-treated (naïve and IL-10) rats demonstrated increased macrophage phagocytosis (**Panel D**), increased production of reactive oxygen species (ROS) (**Panel E**), and increased macrophage phagocytic index (**Panel F**). Macrophages isolated from lungs of IL-10-UC-MSC-treated rats demonstrated greater phagocytic indices than did macrophages from naïve-UC-MSC-treated rats (**Panel F**), *N* = 5/group; * *p* < 0.05 vs. PBS group; & *p* < 0.05 vs. Reg UC-MSCs-treated group (8–10 randomly chosen fields/slide were done in duplicate, quantified, and taken as one sample).

Alveolar macrophages isolated from *E. coli* pneumonia rats treated with naïve and IL-10 UC-MSCs demonstrated greater phagocytosis ex vivo than did macrophages from vehicle-treated rats (Figure 5A–C). Quantitative analysis confirmed that macrophages isolated from lungs of (naïve and IL-10) UC-MSC-treated rats demonstrated increased macrophage phagocytosis (Figure 5D), increased production of reactive oxygen species (ROS) (Figure 5E), and increased macrophage phagocytic index (Figure 5F). Macrophages isolated from lungs of IL-10-UC-MSC-treated rats demonstrated greater phagocytic indices than macrophages from naïve-UC-MSC-treated rats (Figure 5F).

**Figure 6:**
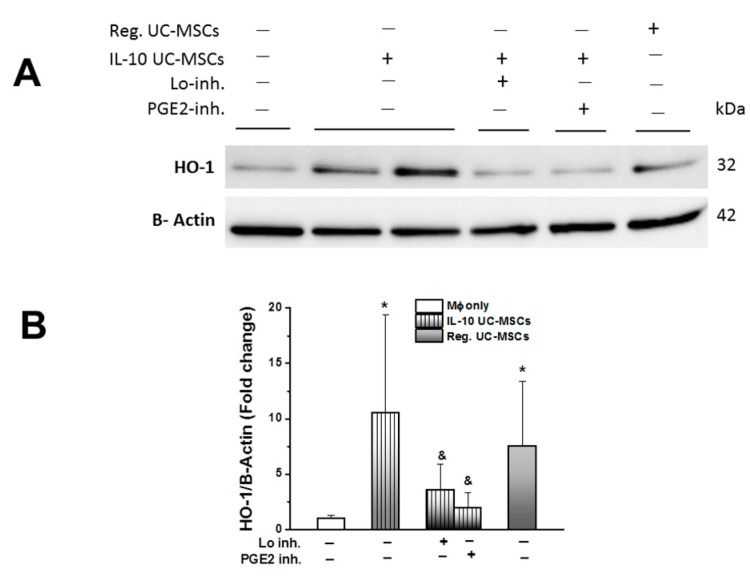
**IL-10 UC-MSCs increase heme oxygenase-1 (HO-1) expression in hMф.** A representative Western blot image (**Panel A**) shows that both IL-10 and naïve UC-MSCs increase HO-1 expression in hMф, while Lo and especially PGE2 inhibitor are able to decrease HO-1 expression induced by IL-10 UC-MSCs. Quantification of four gels confirmed these findings (**Panels A–B**). *N* = 5–7/group; * *p* < 0.05 vs. hMф group; & *p* < 0.05 vs. Mф + IL-10 UC-MSCs.

Subsequent studies demonstrated that exposure to IL-10 UC-MSCs increased heme oxygenase-1 (HO-1) concentrations in human blood monocyte-derived macrophages (Figure 6A). This increase in HO-1 was blocked by inhibitors of prostaglandin E2 and lipoxygenase (Figure 6B).

**Figure 7:**
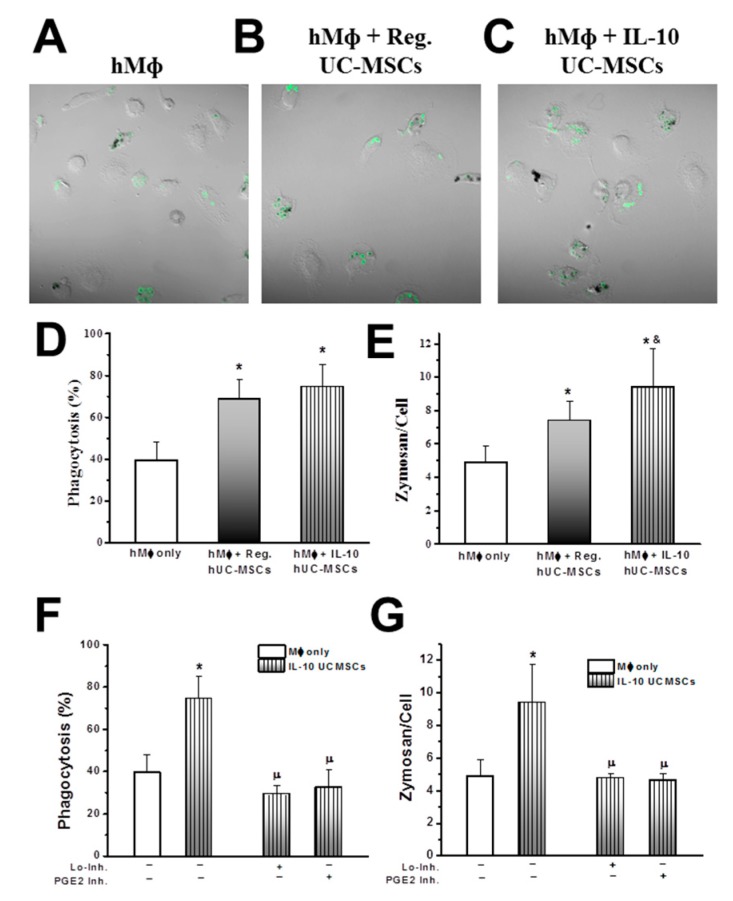
**IL-10 UC-MSCs increase the phagocytic index of human Mф.** Representative images (**A**–**C**), show that both regular and IL-10 UC-MSCs increase phagocytosis of hMф (**D**), while the phagocytic index is further increased by IL-10 UC-MSCs (**E**). The effects of IL-10 UC-MSCs on phagocytosis (**F**) and the phagocytic index (**G**) were blocked by Lo-inhibitors and by prostaglandin E2 inhibitors. *N* = 5–7/group; * *p* < 0.05 vs. hMф group, & *p* < 0.05 vs. Mф + Reg. UC-MSCs, µ *p* < 0.05 vs. Mф + IL-10 UC-MSCs.

Further studies demonstrated that IL-10 overexpressing UC-MSCs can enhance phagocytosis in human macrophages, significantly increasing the phagocytosis index (Zymosan particles/cell) compared to that of naïve-UC-MSCs-treated macrophages (Figure 7A–E). The effects of IL-10 UC-MSCs on phagocytosis (Figure 7F) and the phagocytic index (Figure 7G) were blocked by LXA4 (Lo-inhibitors) or prostaglandin E2 inhibition.

## 4. Discussion

These studies provide a number of novel and important insights. Firstly, our findings demonstrate the therapeutic potential of IL-10 overexpressing UC-MSCs in a clinically relevant model of live bacterial (*E. coli*)-induced pneumonia. While naïve UC-MSCs reduced the severity of *E. coli* pneumosepsis, the efficacy was enhanced with IL-10 overexpressing UC-MSCs. Prior demonstrations of the efficacy of this approach have been confined to sterile injury models. Second, we provided insights into key effects of IL-10 overexpression on UC-MSCs in the setting of pneumonia-induced lung injury. IL-10 overexpressing MSCs were more effective in reducing bacterial load in the lung, reducing *E. coli*-induced lung dysfunction, and decreasing histologic lung injury. Third, we provided mechanistic insights into the actions of IL-10 overexpressing MSCs, namely, the enhancement of macrophage phagocytosis and production of ROS, which are necessary for the killing of phagocytosed bacteria. Taken together, our demonstration that IL-10 overexpression in UC-MSCs can enhance their therapeutic effects in a clinically relevant infection model and can enhance human macrophage function via this mechanism suggests that this approach deserves further exploration as a potential therapy for infection-induced ARDS.

MSCs demonstrate substantial therapeutic promise in preclinical models of pneumonia, ARDS, and sepsis [3,4,5,6,24,25,26,27]. The key relevant mechanisms of action of MSCs include decreased alveolar–capillary barrier permeability [6], enhanced alveolar fluid clearance [28], mitochondrial transfer [27,29], and enhancement of injury resolution and tissue repair [7,8,30]. The spectrum of potentially relevant mechanisms of action of MSCs in the setting of microbial tissue injury and reparative processes involves both cell-contact-dependent effects [27,29] and cell-independent effects, i.e., those mediated by paracrine secreted products [4,5] and by exosomes and microvesicles [31] generated by MSCs.

The potential for MSCs to favorably modulate the immune response to bacterial infection and tissue injury is a key focus. MSCs have been demonstrated to enhance macrophage function [24] in response to infection, resulting in greater bacterial phagocytosis and killing [3,6,25], reducing tissue bacterial loads, and facilitating injury resolution and tissue repair [4,5]. An important area of investigation is the potential to enhance the immunomodulatory effects of MSCs to further increase their therapeutic potential. “Pre-activation” strategies take advantage of a key attribute of MSCs, namely, their potential to modulate their activity in response to their microenvironment. By pre-exposing the MSCs to cues, such as cytokines that are found in the injury microenvironment, MSCs can be activated. Our group recently demonstrated the potential for interferon-γ-preactivated MSCs to enhance the therapeutic effect of microvesicles from these cells in *E. coli* pneumonia [31].

Another promising strategy is to overexpress genes on MSCs to enhance their efficacy. Overexpression of C-X-C chemokine receptor type 4 (CXCR4) on MSCs has been demonstrated to increase MSC homing to sites of injury [32]. Overexpression of immune modulators on MSCs offers the potential to use MSCs as a “delivery” strategy, given their potential to home to sites of injury [9]. IL-10 is a key immunoregulatory cytokine that can suppress potentially damaging pro-inflammatory responses and which aids the resolution and restoration of homeostasis in both ARDS and sepsis [10]. Direct overexpression of IL-10 has been demonstrated to reduce ischemia–reperfusion injury in lung transplant models [12]. Prior studies of overexpression of IL-10 in MSCs demonstrated enhanced MSC therapeutic efficacy in preclinical models of endotoxin-induced [13] and ischemia–reperfusion-induced [14] lung injury and following myocardial infarction [15]. The effects of IL-10 overexpressing MSCs in the setting of live bacterial infection are not known.

In the current studies, the overexpression of IL-10 on UC-MSCs did not adversely affect the MSC phenotype or viability and resulted in substantially increased UC-MSC secretion of IL-10, demonstrating the efficacy of the transduction approach. These IL-10 overexpressing MSCs demonstrated a number of potentially important advantages over naïve UC-MSCs in our model of *E. coli*-induced pneumonia. These cells increased the proportion of alveolar macrophages in the alveolus—of importance given the central role of alveolar macrophages in bacterial clearance. IL-10 overexpressing MSCs were more effective in reducing bacterial load in the lung, reducing *E. coli*-induced lung dysfunction, and decreasing histologic lung injury. Lung bacterial counts were not decreased, possibly due to the degree of data variability and to a “survivor bias” where vehicle-treated animals with higher bacterial counts did not survive. The data presented provide novel mechanistic insights into the actions of IL-10 overexpressing MSCs, namely, the enhancement of macrophage phagocytosis and production of ROS, which are necessary for the killing of phagocytosed bacteria. These effects on macrophages appear to be mediated via increases in HO-1, mediated in turn via MSC secretion of lipoxin A4 and prostaglandin E2, consistent with prior reports from our group [24]. We also demonstrated that IL-10 overexpressing UC-MSCs can enhance phagocytosis in human macrophages, which constitutes an important step towards clinical translation of these findings. This in vitro immunomodulation assay of MSC-enhanced macrophage phagocytosis may be a useful assay for MSC potency. Taken together, our demonstration that IL-10 overexpressing UC-MSCs can enhance therapeutic effects in a clinically relevant infection model suggests that this approach deserves further exploration as a potential therapy for infection-induced ARDS.

There are some limitations to these studies that should be considered. First, while these studies were conducted in relevant preclinical models of *E. coli*-induced acute lung injury, caution is required in extrapolating to the clinical condition of ARDS. Studies in larger animals may provide additional insights. Second, we studied a single dose and administration route of IL-10 overexpressing MSCs. The dosage regimen was based on prior studies from our group demonstrating that this was an effective regimen in *E. coli* lung injury [6,25]. We utilized the intravenous route of administration due to prior studies from our group and others demonstrating that this route is as at least as effective as other, more invasive routes such as intrapulmonary or intraperitoneal administration [5,8]. Additional dose–response studies, and studies using differing routes of administration and additional doses, would provide further insights regarding these IL-10 overexpressing UC-MSCs. Third, we did not use UC-MSCs overexpressing a non-functional gene in these studies. We considered it unlikely that the effects of Il-10 overexpression would be a non-specific effect, given that we demonstrated (Figure S2) that the IL-10 overexpressing UC-MSCs produce high quantities of human IL-10 and the fact that the effects seen on macrophage function are consistent with the known effects of IL-10. We did not assess UC-MSC engraftment in the lung as this is not considered to be an important mechanism of action of MSCs. Lastly, characterizing additional mechanisms by which IL-10 overexpressing UC-MSCs may attenuate *E. coli*-induced lung injury will be important to understanding their underlying therapeutic mechanisms.

## 5. Conclusions

We report that IL-10 overexpressing UC-MSCs have enhanced capacity to attenuate *E. coli*-induced lung injury compared to naïve UC-MSCs. A key mechanism of action appears to be mediated via the enhancement of macrophage phagocytosis and macrophage killing of *E. coli*. The demonstration that IL-10 overexpression can enhance UC-MSCs’ therapeutic effects in a clinically relevant infection model and enhance human macrophage function via this mechanism suggests that this approach deserves further exploration as a potential therapy for infection-induced ARDS.

## Supplementary Materials

The following are available online at https://www.mdpi.com/2077-0383/8/6/847/s1, Figure S1: Surface marker profile of fresh and frozen IL-10 overexpressing UC-MSCs vs. naïve (unmodified) UC-MCSs. Figure S2: IL-10 production in naïve MCSs and in IL-10 UC-MSCs, Figure S3: Images of IL-10 overexpressing MSCs vs. naïve (unmodified) MCSs.

## References

[1] Keane C., Jerkic M., Laffey J.G. (2017). Stem cell-based therapies for sepsis. Anesthesiology.

[2] Laffey J.G., Matthay M.A. (2017). Fifty years of research in ARDS: Cell-based therapy for acute respiratory distress syndrome, biology and potential therapeutic value. Am. J. Respir. Crit. Care Med..

[3] Curley G.F., Jerkic M., Dixon S., Hogan G., Masterson C., O’Toole D., Devaney J., Laffey J.G. (2017). Cryopreserved, xeno-free human umbilical cord mesenchymal stromal cells reduce lung injury severity and bacterial burden in rodent *Escherichia coli*-induced acute respiratory distress syndrome. Crit. Care Med..

[4] Curley G., Hayes M., Ansari B., Shaw G., Ryan A., Barry F., O’Brien T., O’Toole D., Laffey J. (2012). Mesenchymal stem cells enhance recovery and repair following ventilator-induced lung injury in the rat. Thorax.

[5] Curley G.F., Ansari B., Hayes M., Devaney J., Masterson C., Ryan A., Barry F., O’Brien T., O’Toole D., Laffey J.G. (2013). Effects of intratracheal mesenchymal stromal cell therapy during recovery and resolution after ventilator-induced lung injury. Anesthesiology.

[6] Devaney J., Horie S., Masterson C., Elliman E., Barry F., O’Brien T., Curley G., Toole D., Laffey J. (2015). Human mesenchymal stromal cells decrease the severity of acute lung injury induced by *E. coli* in the rat. Thorax.

[7] Hayes M., Curley G.F., Masterson C., Devaney J., O’Toole D., Laffey J.G. (2015). Mesenchymal stromal cells are more effective than the MSC secretome in diminishing injury and enhancing recovery following ventilator-induced lung injury. Intensive Care Med. Exp..

[8] Hayes M., Masterson C., Devaney J., Barry F., Elliman S., O’Brien T., O’Toole D., Curley G.F., Laffey J.G. (2015). Therapeutic efficacy of human mesenchymal stromal cells in the repair of established ventilator-induced lung injury in the rat. Anesthesiology.

[9] Oggu G.S., Sasikumar S., Reddy N., Ella K.K.R., Rao C.M., Bokara K.K. (2017). Gene delivery approaches for mesenchymal stem cell therapy: Strategies to increase efficiency and specificity. Stem Cell Rev..

[10] Zhao H.Q., Li W.M., Lu Z.Q., Sheng Z.Y., Yao Y.M. (2015). The growing spectrum of anti-inflammatory interleukins and their potential roles in the development of sepsis. J. Interferon Cytokine Res..

[11] Li H.D., Zhang Q.X., Mao Z., Xu X.J., Li N.Y., Zhang H. (2015). Exogenous interleukin-10 attenuates hyperoxia-induced acute lung injury in mice. Exp. Physiol..

[12] Machuca T.N., Cypel M., Bonato R., Yeung J.C., Chun Y.M., Juvet S., Guan Z., Hwang D.M., Chen M., Saito T. (2017). Safety and efficacy of ex vivo donor lung adenoviral IL-10 gene therapy in a large animal lung transplant survival model. Hum. Gene Ther..

[13] Wang C., Lv D., Zhang X., Ni Z.A., Sun X., Zhu C. (2018). Interleukin-10-overexpressing mesenchymal stromal cells induce a series of regulatory effects in the inflammatory system and promote the survival of endotoxin-induced acute lung injury in mice model. DNA Cell Biol..

[14] Manning E., Pham S., Li S., Vazquez-Padron R.I., Mathew J., Ruiz P., Salgar S.K. (2010). Interleukin-10 delivery via mesenchymal stem cells: A novel gene therapy approach to prevent lung ischemia-reperfusion injury. Hum. Gene Ther..

[15] Meng X., Li J., Yu M., Yang J., Zheng M., Zhang J., Sun C., Liang H., Liu L. (2018). Transplantation of mesenchymal stem cells overexpressing IL10 attenuates cardiac impairments in rats with myocardial infarction. J. Cell Physiol..

[16] Sarugaser R., Lickorish D., Baksh D., Hosseini M.M., Davies J.E. (2005). Human umbilical cord perivascular (HUCPV) cells: A source of mesenchymal progenitors. Stem Cells.

[17] Sarugaser R., Ennis J., Stanford W.L., Davies J.E. (2009). Isolation, propagation, and characterization of human umbilical cord perivascular cells (HUCPVCs). Methods Mol. Biol..

[18] O’Croinin D.F., Nichol A.D., Hopkins N., Boylan J., O’Brien S., O’Connor C., Laffey J.G., McLoughlin P. (2008). Sustained hypercapnic acidosis during pulmonary infection increases bacterial load and worsens lung injury. Crit. Care Med..

[19] Costello J., Higgins B., Contreras M., Chonghaile M.N., Hassett P., O’Toole D., Laffey J.G. (2009). Hypercapnic acidosis attenuates shock and lung injury in early and prolonged systemic sepsis. Crit. Care Med..

[20] Higgins B.D., Costello J., Contreras M., Hassett P., O’Toole D., Laffey J.G. (2009). Differential effects of buffered hypercapnia versus hypercapnic acidosis on shock and lung injury induced by systemic sepsis. Anesthesiology.

[21] Laffey J.G., Honan D., Hopkins N., Hyvelin J.M., Boylan J.F., McLoughlin P. (2004). Hypercapnic acidosis attenuates endotoxin-induced acute lung injury. Am. J. Respir. Crit. Care Med..

[22] Jerkic M., Peter M., Ardelean D., Fine M., Konerding M.A., Letarte M. (2010). Dextran sulfate sodium leads to chronic colitis and pathological angiogenesis in Endoglin heterozygous mice. Inflamm. Bowel Dis..

[23] Canton J., Khezri R., Glogauer M., Grinstein S. (2014). Contrasting phagosome pH regulation and maturation in human M1 and M2 macrophages. Mol. Biol. Cell.

[24] Rabani R., Volchuk A., Jerkic M., Ormesher L., Garces-Ramirez L., Canton J., Masterson C., Gagnon S., Tatham K.C., Marshall J. (2018). Mesenchymal stem cells enhance NOX2-dependent reactive oxygen species production and bacterial killing in macrophages during sepsis. Eur. Respir. J..

[25] Masterson C., Devaney J., Horie S., O’Flynn L., Deedigan L., Elliman S., Barry F., O’Brien T., O’Toole D., Laffey J.G. (2018). Syndecan-2-positive, bone marrow-derived human mesenchymal stromal cells attenuate bacterial-induced acute lung injury and enhance resolution of ventilator-induced lung injury in rats. Anesthesiology.

[26] Krasnodembskaya A., Song Y., Fang X., Gupta N., Serikov V., Lee J., Matthay M. (2010). Antibacterial effect of human mesenchymal stem cells is mediated in part from secretion of the antimicrobial peptide LL-37. Stem Cells.

[27] Jackson M.V., Morrison T.J., Doherty D.F., McAuley D.F., Matthay M.A., Kissenpfennig A., O’Kane C.M., Krasnodembskaya A.D. (2016). Mitochondrial transfer via tunneling nanotubes is an important mechanism by which mesenchymal stem cells enhance macrophage phagocytosis in the in vitro and in vivo models of ARDS. Stem Cells.

[28] McAuley D.F., Curley G.F., Hamid U.I., Laffey J.G., Abbott J., McKenna D.H., Fang X., Matthay M.A., Lee J.W. (2014). Clinical grade allogeneic human mesenchymal stem cells restore alveolar fluid clearance in human lungs rejected for transplantation. Am. J. Physiol Lung Cell Mol. Physiol..

[29] Islam M.N., Das S.R., Emin M.T., Wei M., Sun L., Westphalen K., Rowlands D.J., Quadri S.K., Bhattacharya S., Bhattacharya J. (2012). Mitochondrial transfer from bone-marrow-derived stromal cells to pulmonary alveoli protects against acute lung injury. Nat. Med..

[30] Fang X., Abbott J., Cheng L., Colby J.K., Lee J.W., Levy B.D., Matthay M.A. (2015). Human mesenchymal stem (stromal) cells promote the resolution of acute lung injury in part through lipoxin A4. J. Immunol..

[31] Varkouhi A., Jerkic M., Ormesher L., Gagnon S., Goyal S., Rabani R., Chen P., Gu F., dos Santos C., Curley G.F. (2019). Extracellular vesicles derived from Interferon-g primed human umbilical cord derived mesenchymal stromal cells demonstrate enhanced efficacy in pneumonia induced ARDS. Anesthesiology.

[32] Chen W., Li M., Cheng H., Yan Z., Cao J., Pan B., Sang W., Wu Q., Zeng L., Li Z. (2013). Overexpression of the mesenchymal stem cell Cxcr4 gene in irradiated mice increases the homing capacity of these cells. Cell Biochem. Biophys..

