# Supplementary material for: Overexpression of IL-10 Enhances the Efficacy of Human Umbilical-Cord-Derived Mesenchymal Stromal Cells in E. coli Pneumosepsis
Source: J Clin Med. 2019 Jun 13;8(6):847. doi: 10.3390/jcm8060847 (PMC6616885; doi:10.3390/jcm8060847)
Supplement: Supplementary file 1 [file jcm-08-00847-s001.pdf]

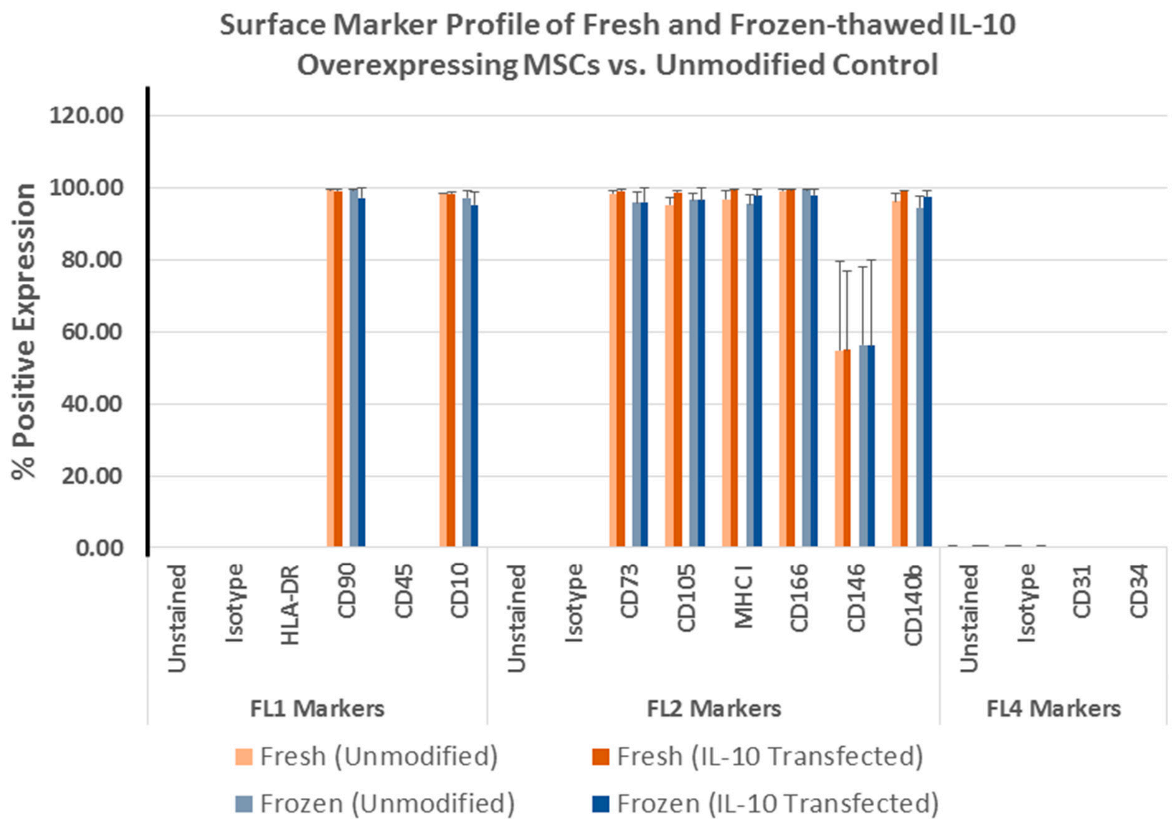

Figure S1. Surface marker profiles of fresh and frozen IL-10 overexpressing MSCs vs. naïve (unmodified) MCSs

There is no difference in surface marker profiles between fresh and frozen IL-10 overexpressing MSCs vs. naïve (unmodified) MCSs.

N = 3 donors/group.

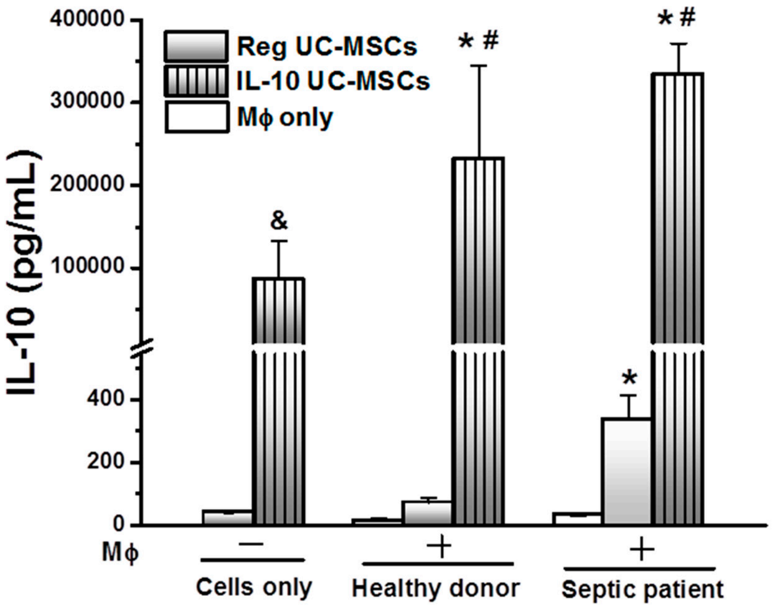

Figure S2. IL-10 production in naïve MCSs and in IL-10 UC-MSCs

Human IL-10 secretion is greatly increased in IL-10 overexpressing UC-MCs compared to naïve MSCs.

Both naïve and IL-10 UC-MSCs result in a marked induction of IL-10 when co-cultured with macrophages (Mϕ) isolated from *septic patients*.

N = 5/group; **p* < 0.05 vs. Mϕ only, #*p* < 0.05 vs. Reg UC-MSCs treated group, &*p* < 0.05 vs. Reg UC-MSCs only.

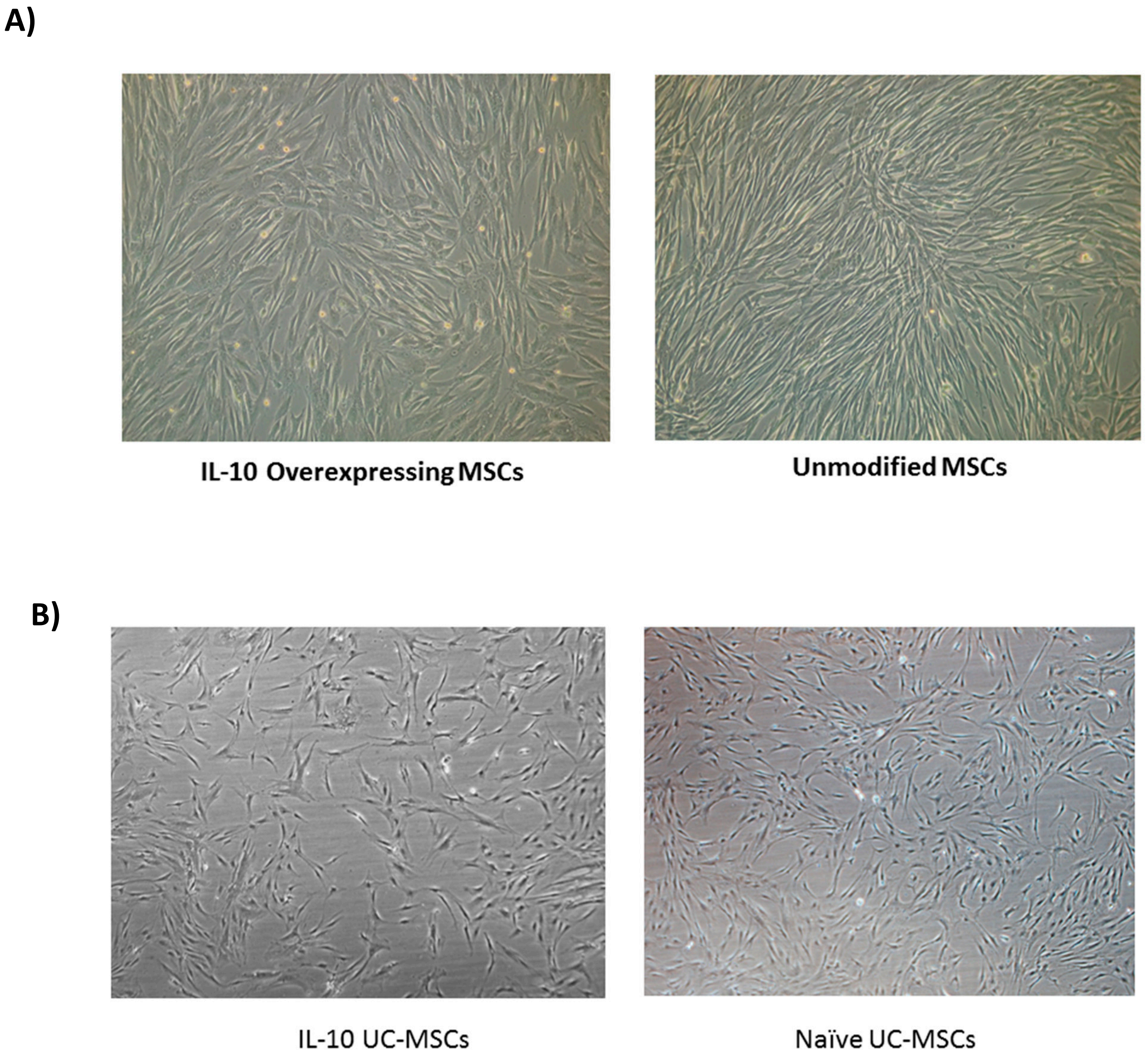

Figure S3. Images of IL-10 overexpressing MSCs vs. naïve (unmodified) MCSs taken with 10× objective after transfection (**A**) and after thawing for the animal treatment (**B**). There is no change in cell morphology in IL-10 transfected cells.
